# The Design and Fabrication of Shear-Mode Piezoelectric Accelerometers with High Bandwidth Using High Piezoelectric g-Coefficient NKN-Based Ceramics

**DOI:** 10.3390/ma18081813

**Published:** 2025-04-15

**Authors:** Jian-Hao Huang, Chien-Min Cheng, Sheng-Yuan Chu, Cheng-Che Tsai

**Affiliations:** 1 Department of Electronic Engineering, Southern Taiwan University of Science and Technology, Tainan 710301, Taiwan; mb130207@stust.edu.tw (J.-H.H.); ccmin@stust.edu.tw (C.-M.C.); 2 Department of Electrical Engineering, National Cheng Kung University, Tainan 70101, Taiwan; 3 Department of Game and Animation Design, Tung Fang Design University, Kaohsiung 829, Taiwan; tsai480116@gmail.com

**Keywords:** lead-free piezoelectric ceramics, solid-state synthesis, sintering aid, piezoelectric effect, shear-type accelerometer

## Abstract

In this work, lead-free (Na_0.475_K_0.475_Li_0.05_)NbO_3_ + *x* wt.% ZnO (NKLN, *x* = 0 to 0.3) piezoelectric ceramics with high piezoelectric g-coefficients were prepared by conventional solid-state synthesis method. By adding different concentrations of ZnO dopants, we aimed to improve the material properties and enhance their piezoelectric properties. The effects of the ZnO addition on the microstructure, dielectric, piezoelectric and ferroelectric properties of the proposed samples are investigated. Adding ZnO reduced the dielectric constant and improved the g-value of the samples. The properties of the samples without ZnO doping were g_33_ = 31 mV·m/N, g_15_ = 34 mV·m/N, k_p_ = 0.39, Q_m_ = 92, ε_r_ = 458, d_33_ = 127 pC/N and dielectric loss = 3.4%. With the preferable ZnO doping of 1 wt.%, the piezoelectric properties improved to g_33_ = 40 mV·m/N, g_15_ = 44 mV·m/N, k_p_ = 0.44, Q_m_ = 89, ε_r_ = 406, d_33_ = 139 pC/N and dielectric loss = 2.4%. Finally, ring-shaped shear mode piezoelectric accelerometers were fabricated using the optimum ZnO-doped samples. The simulated resonant frequency using ANSYS 2024 R1 software was approximately 23 kHz, while the actual measured resonant frequency of the devices was 19 kHz. The sensitivity was approximately 7.08 mV/g. This piezoelectric accelerometer suits applications requiring lower sensitivity and higher resonant frequencies, such as monitoring high-frequency vibrations in high-speed machinery, robotic arms or scientific research and engineering fields involving high-frequency vibration testing.

## 1. Introduction

With the advances in technology, automated production equipment is gradually becoming popular to improve production efficiency and reduce costs. The rapid development of the Internet of Things (IoT) has brought about a full range of information exchange and community sharing. The smart factory has flourished under this trend, combining the IoT, digitization systems, big data analytics and automation technologies. The smart factory connects all aspects of the manufacturing process in a data-driven manner, enabling higher production efficiency, flexible configurations and better-quality standards. In this process, the Internet of Things (IoT) provides real-time data monitoring, automation control and fault prediction for production equipment through various sensing elements, thus realizing the intelligence and automation of the production process. Sensors play a key role in this system, converting real-world data into digital signals, which can be analyzed and optimized for intelligent automation [1].

Currently, commercially available piezoelectric sensing devices typically use lead zirconate titanate (PZT) as the material due to its excellent piezoelectric properties and temperature stability [2,3,4]. However, in recent years, with the rise of environmental awareness, industrial countries such as the European Union and Japan have banned using lead-containing materials to prevent environmental contamination by heavy metals. Although the European Union and Japan have not completely banned the use of lead, the EU initially introduced the Restriction of Hazardous Substances Directive 2002/95/EC (RoHS) in 2002, while Japan implemented the Chemical Substance Control Law (CSCL) in 1973 to regulate the use of lead. Consequently, lead-free materials have garnered significant attention, with common examples including barium titanate (BaTiO_3_), bismuth sodium titanate ((Bi, Na)TiO_3_) and sodium potassium niobate (NaKNbO_3_) [5,6,7]. Among these materials, sodium potassium niobate series materials are noted for their outstanding electromechanical coupling coefficient (k^2^) and high Curie temperature (Tc), approximately 0.48 and 470 °C [8], making them suitable for various component operating environments. This has led to significant research interest and development in lead-free ceramics. However, the sintering condition and synthesis process of sodium potassium niobate materials have been challenging due to the volatilization of sodium and potassium ions at high temperatures, leading to alkali metal vacancies and oxygen vacancies. As a result, achieving high density and maintaining the piezoelectric properties of the material have been difficult [9,10]. To address this issue, many researchers have explored doping with other elements to improve sintering density and overcome the problem of hygroscopicity in NKN-based systems. Previous reports have indicated that doping with Li, Ta and Sb ions in NKN-based (Na, K, Li)(Nb, Sb, Ta)O_3_ can increase the formation of polycrystalline phase boundaries and decrease alkali metal vacancies at room temperature [11,12,13,14]. Past research has also demonstrated that adding sintering aids such as ZrO_2_, CuO, MnO, LiF, CuF_2_, SnO_2_ and ZnO can effectively lower the sintering temperature [15,16,17]. Among these dopants, ZnO has been used in the PZT system to improve Tc, enhance the temperature stability and decrease the dielectric properties [18]. Li et al. also reported that ZnO was found to exhibit a donor doping effect in a 0.92(K_0.5_Na_0.5_)NbO_3_-0.02(Bi_0.5_Li_0.5_)TiO_3_-0.06BaZrO_3_ system, significantly enhancing the ferroelectricity and piezoelectricity, accompanied by a significant contribution to the suppressed formation of oxygen vacancy. [19]

In addition to material development, exploring suitable applications is also a crucial direction. Piezoelectric accelerometers are essential for detecting vibrations in machinery and other structures [20]. These sensors are commonly used to monitor the condition and safety of aerospace and automotive components and various industrial facilities. There are two other types of accelerometers besides the shear mode. One is the compression type. It consists of an upper mass block and a lower piezoelectric sheet. When the mass is extruded and deformed by an external force, the lower piezoelectric sheet generates a charge signal output. This simple and rigid structure results in a high resonance frequency. However, this design is susceptible to bottom strain and thermal effects, which may limit applications in unstable environments. The other is the flexural type. Its cantilevered beam structure is commonly used, where the mass block causes lateral stretching and compression of the cantilevered beam when an external force is applied to it, which in turn causes bending and deformation of the piezoelectric material and ultimately generates the charge signal output. This structure is characterized by relatively low stiffness and can therefore be used in lower frequency measurement environments and is particularly suitable for sensing small vibrations. Although it is less susceptible to thermal effects, its overall size is relatively large, and it is susceptible to damage. In this work, our shear accelerometer has a high g value, g_33_ = 40 mV·m/N, g_15_ = 44 mV·m/N, the sensitivity is about 7.08 mV/g and available frequencies are approximately 100~12 kHz. This piezoelectric accelerometer benefits from less bottom strain and thermal effects, as well as high stiffness and usable bandwidth, providing more sensitive sensing performance. Compared to MEMS technology, ceramics synthesized by traditional oxide methods offer advantages such as shorter development cycles, simpler manufacturing and large-scale production capabilities. Lead-free shear-mode piezoelectric accelerometers, in particular, have the advantage of stability in temperature variation and high-frequency response characteristics, making them especially suitable for factory environments with dramatic temperature changes. Furthermore, this design minimizes interference from mechanical vibrations introduced by the base or environmental vibrations, enhancing their resonance frequency and noise resistance.

Materials with a high piezoelectric g_15_ coefficient typically generate a significant amount of voltage under relatively small mechanical stress or strain, indicating that the material possesses greater mechanical stiffness. Consequently, when such materials are used in a system, they can effectively enhance the overall stiffness of the system, thereby increasing its resonance frequency. To achieve this, the solid-state synthesis method was used to prepare (Na_0.475_K_0.475_Li_0.05_)NbO_3_ ceramics based on Ref. [21] and *x* wt.% ZnO (*x* = 0–0.3) were added, aiming to enhance the piezoelectric g-value through lower dielectric constants. This approach led to the development of a simple and cost-effective piezoelectric shear-mode accelerometer, which could serve as a replacement for lead-containing accelerometers.

## 2. Experimental Procedures

In this study, piezoelectric ceramics were prepared using the traditional solid-state reaction method. (Na_0.475_K_0.475_Li_0.05_)NbO_3_ + *x* wt.% ZnO (*x* = 0–0.3) (named NKLN-*x*Z)ceramic powders were first prepared by weighing Na_2_CO_3_, K_2_CO_3_, Li_2_CO_3_ and Nb_2_O_5_ according to the molar ratios, then adding them into a ball mill with anhydrous ethanol and zirconia balls for 24 h of milling. After separating the slurry from the zirconia balls, it was dried in an 80 °C oven. The dried powders were then ground and sieved through a 50-mesh screen, after which it was placed in an alumina crucible, covered and calcined. The calcination was conducted with a heating rate of 5 °C/min up to 850 °C, holding at that temperature for 5 h. After calcination, the powder was crushed and weighed, then mixed with *x* wt.% ZnO, where *x* = 0.1, 0.2, 0.3. The mixture was added to a ball mill with anhydrous ethanol and zirconia balls for a second 24 h of milling. After separating the slurry from the zirconia balls, it was dried again in an 80 °C oven. The dried powders were then ground and sieved through a 100-mesh screen to achieve a uniform particle size.

Following these steps, the ceramic powders were mixed with 15% PVA binder and thoroughly combined before being dried in an 80 °C oven for 1 h. The dried powders were then ground and sieved through a 100-mesh screen to improve powder flowability for granulation. After granulation, the powders were placed into a circular mold and pressed using a hydraulic press to form green bodies with a diameter of 15 mm and a thickness of 1.4 mm. These green bodies were then placed in a high-temperature furnace to remove the PVA binder at 650 °C for 6 h. Following binder removal, the samples were sintered in air at 1030–1070 °C for 4 h. After sintering, the samples were polished sequentially with different grades of sandpaper to achieve a smooth surface. After cleaning, silver paste was applied to both sides of the samples as conductive electrodes. The samples were then placed in a silicone oil bath for polarization under a DC high-voltage electric field to develop piezoelectric properties. The polarization field was 30 kV/cm, and the polarization temperature was 60 °C. After polarization, the samples were removed from the silicone oil bath, cleaned with alcohol and left to rest in a constant-temperature chamber for one day to stabilize the internal electric dipoles. Once stabilized, the electrical properties of the samples were measured.

The crystal structure of the sintered samples was examined using X-ray diffraction (XRD, D2 PHASER, Bruker, Berlin, Germany) with Cu Kα radiation (λ = 1.5416 Å). Global simulations of the complete XRD patterns were performed using the TOPAS v4.2 software to refine the lattice parameters. The microstructure was observed using a scanning electron microscope (SEM, SU8000, Hitachi, Tokyo, Japan). Dielectric properties were measured over a 0 to 400 °C temperature range using an LCR meter (E4980A, Agilent, Santa Clara, CA, USA). Ferroelectric properties were characterized by measuring hysteresis loops at elevated temperatures using a multiferroic and ferroelectric testing system (Precision Multiferroic II Ferroelectric Tester, Radiant Technologies, Inc., Albuquerque, NM, USA). The samples were polarized for 30 min at 60 °C and 30 kV/cm DC field to induce piezoelectric properties. Piezoelectric properties were measured using a quasi-static d_33_ meter (90-20301 d_33_ Meter, APC, West Kingston, RI, USA) and an impedance analyzer (E4990A, Keysight, Santa Rosa, CA, USA). The piezoelectric constants were determined using the d_33_ meter (90-20301 d_33_ Meter, APC, USA). The electromechanical coupling coefficient (k_p_) at room temperature was measured using resonance and anti-resonance methods according to IEEE standards.

## 3. Results and Discussion

**Figure 1:**
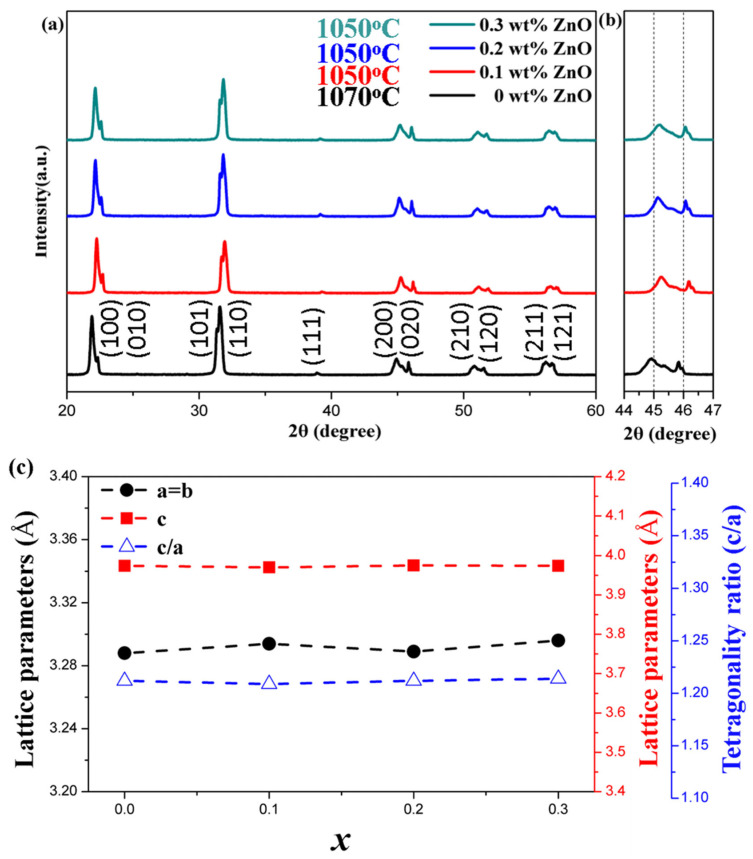
Different *x* ratios of NKLN + *x* wt.% ZnO (*x* = 0 to 0.3). (**a**) XRD diffraction patterns in the range of 20° to 60°; (**b**) XRD diffraction patterns in the range of 44° to 47°; (**c**) variation in lattice parameters and tetragonality ratio (c/a) with ZnO content.

Figure 1a shows the X-ray diffraction (XRD) patterns for NKLN-*x*Z samples (*x* = 0 to 0.3) in the 20° to 60° range. The sample with 0 wt.% ZnO was sintered at 1070 °C, the sample with 0.1 wt.% ZnO at 1050 °C and the remaining samples were sintered at 1030 °C. The materials exhibit a typical perovskite structure with no significant second phases. In the orthorhombic phase of KNN, the peak at 45 degrees should have a higher intensity than the peak at 46 degrees, as seen in Figure 1a. In the tetragonal phase of KNN, the peak at 46 degrees should have a higher intensity than the peak at 45 degrees, but this is not seen in any of the samples. According to the literature, the characteristic phase structure peaks of NKN series materials are determined based on the relative intensities of peaks around 40° to 50° in the XRD patterns, as shown in Figure 1b. The characteristic phase structure peaks are located at angles between 44° and 46.5°. The ionic radius of Zn is 0.74 Å while that of Na and K is 1.39 Å and 1.64 Å, respectively. With increasing *x* content to 0.1, both (200) and (020) peaks gradually shift to higher angles, meaning Zn ions entered the A-site. Further increasing the ZnO doping concentration, some Zn ions entered the B-site to shift the peak to lower angles. Figure 1c shows the lattice constant c decreases with the increase in ZnO content, leading to a reduction in lattice volume. Consequently, the characteristic peaks shift toward higher angles. The tetragonality ratio c/a in Figure 1c first decreases and then increases with the increase in ZnO content. This structural evolution can be attributed to several factors: the incorporation of Zn^2+^ cations into the perovskite structure, the presence of polymorphism observed in KNLN-based materials and a significant decrease in density when *x* > 0.2 wt.% is added to this material.

**Figure 2:**
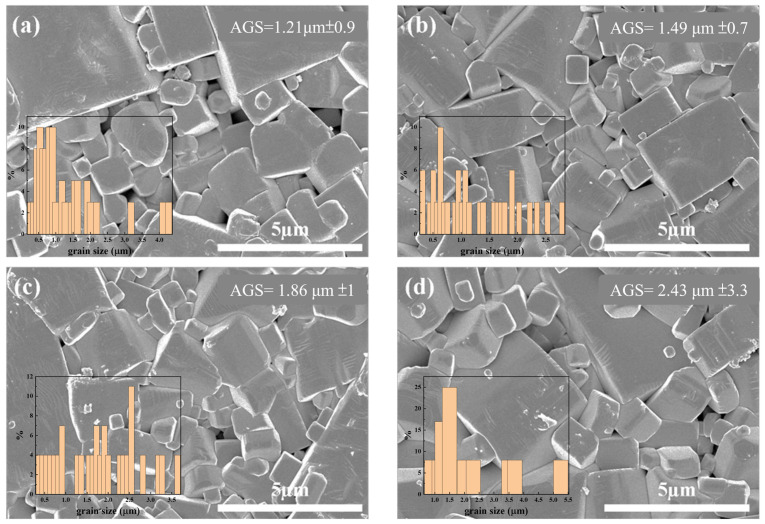
SEM surface structures of NKLN + *x* wt.% ZnO (*x* = 0 to 0.3): (**a**) *x* = 0; (**b**) *x* = 0.1; (**c**) *x* = 0.2; (**d**) *x* = 0.3.

**Figure 3:**
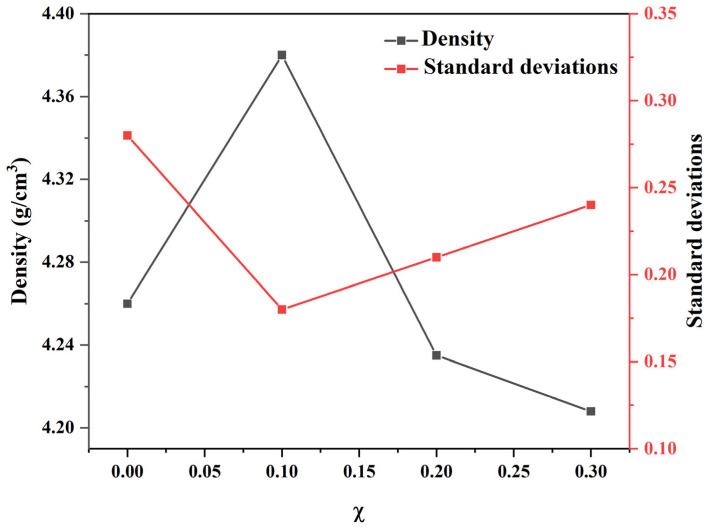
Density chart and standard deviations of NKLN + *x* wt.% ZnO (*x* = 0 to 0.3).

The surface microstructures of all samples were observed using scanning electron microscope (SEM)-simple image view. In Figure 2a–d, it can be seen that the ceramic micrograins mainly exhibit rectangular and cubic shapes. ZnO dopants were found to facilitate grain growth. Figure 3 shows that preferable ZnO doping (0.1 wt.%) increases the surface contact between grains, improving material density, as shown in Figure 3. This is because ZnO acts as a flux in this work. When doped at 0.1 wt.%, it reaches the optimal doping level, increasing the sample density. However, excessive doping prevents ZnO from integrating into the main structure, resulting in a decrease in density.

**Figure 4:**
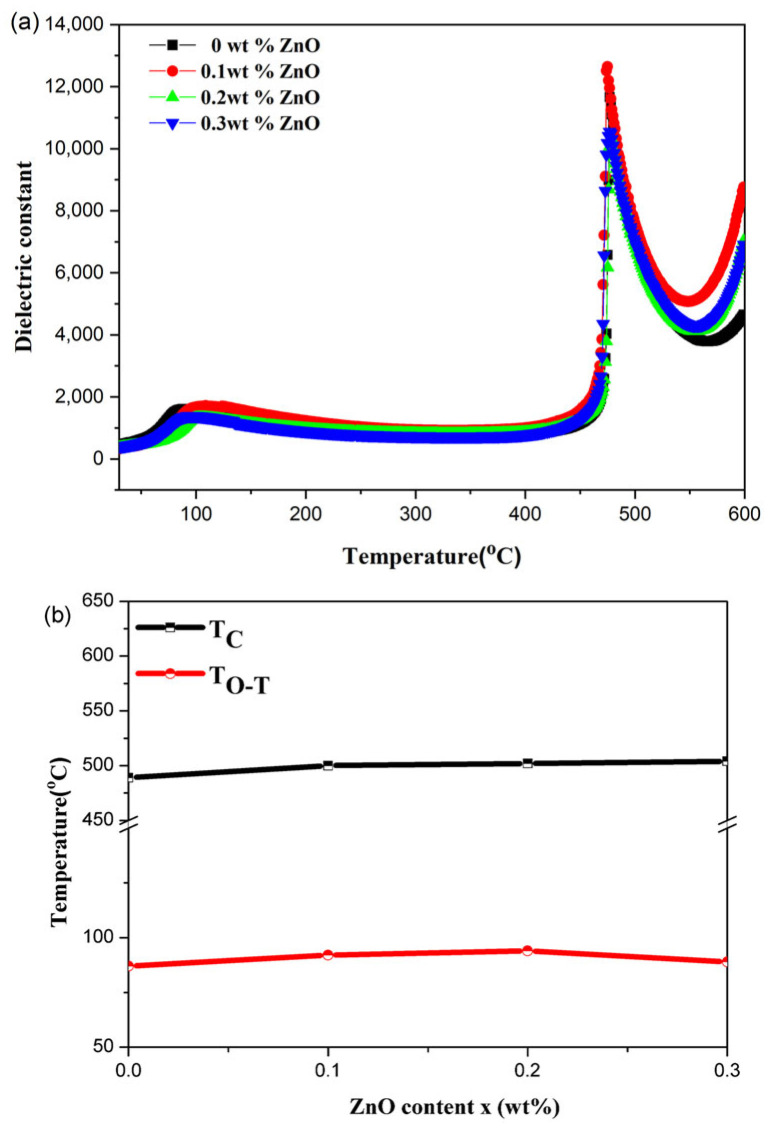
(**a**) Temperature-dependent dielectric constant for different *x* ratios of NKLN + *x* wt.% ZnO (*x* = 0 to 0.3), with (**b**) showing the trend in phase transition temperature points.

Figure 4a shows the temperature-dependent dielectric constant (at 1 kHz) for all samples. It can be observed that the Curie temperature (Tc) increases almost linearly with the addition of ZnO, which is consistent with the changes in unit cell dimensions and indicates that Zn^2+^ substitution at A-sites raises Tc [22]. Figure 4b displays a secondary transition within the 50–100 °C temperature range, corresponding to the orthorhombic to tetragonal phase transition (T_O-T_). The temperature at which this phase transition occurs increases with ZnO content but decreases after reaching 0.3 wt.% ZnO. As ZnO content increases, the transition temperature rises, indicating the presence of polymorphism phase in the T_O-T_ region [23,24].

**Figure 5:**
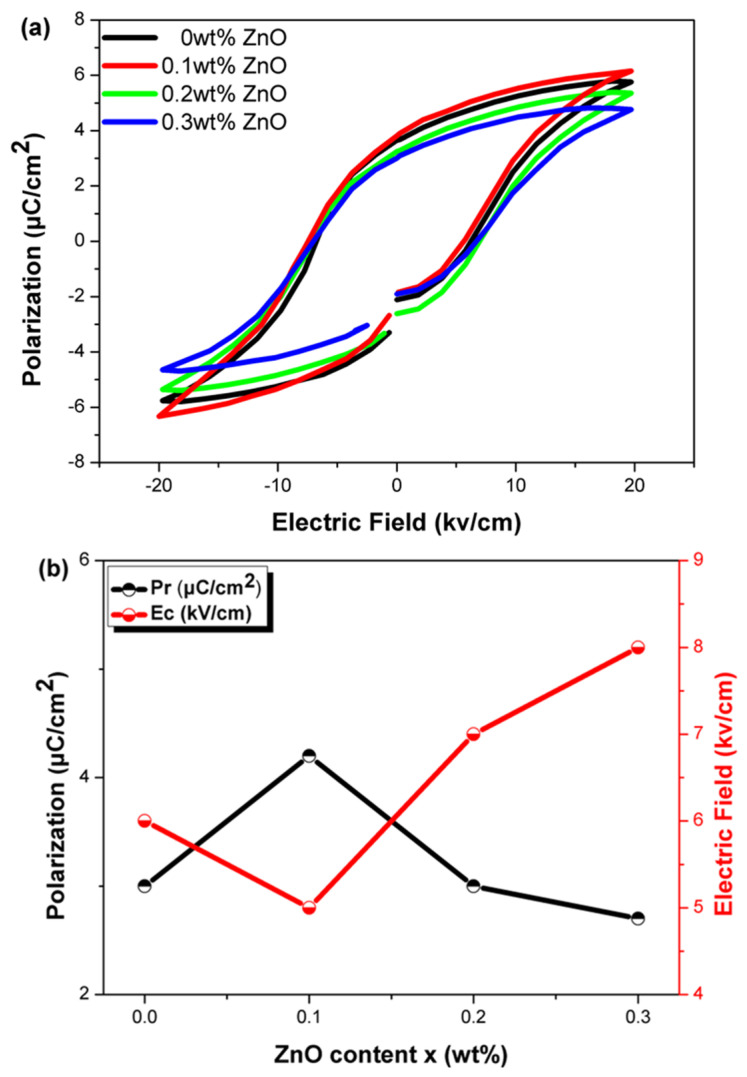
(**a**) Ferroelectric hysteresis loops of NKLN + *x* wt.% ZnO (*x* = 0 to 0.3) measured at room temperature under an electric field of 20 kV/cm, and (**b**) the variations in remanent polarization (Pr) and coercive field (Ec).

Figure 5a shows the ferroelectric hysteresis loops of the samples measured at room temperature under an electric field of 20 kV/cm. It can be observed that with increasing ZnO content, the hysteresis loops gradually rise. Figure 5b indicates that at *x* = 0.1, the material exhibits the highest Pr value and the lowest Ec value. When the ZnO content is low, Ec decreases slightly, possibly due to the reduction in oxygen vacancies that stabilize the domain wall movement. This occurs because Zn^2+^ donor ions (A-site substitution) reduce oxygen vacancies to maintain charge neutrality [22].

**Figure 6:**
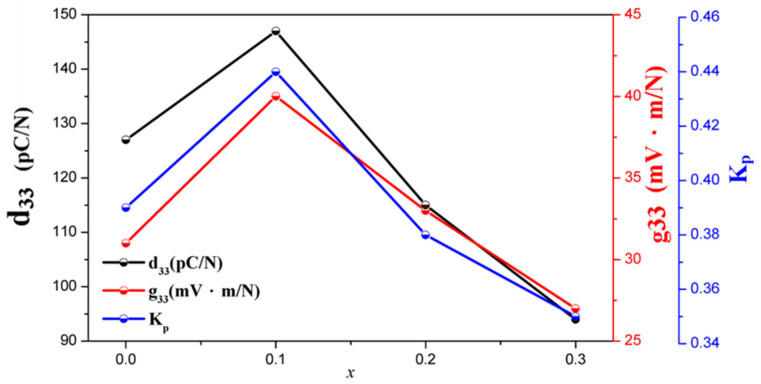
Piezoelectric properties of NKLN + *x* wt.% ZnO (*x* = 0 to 0.3), including d_33_, g_33_ and k_p_.

Figure 6 shows the trends in piezoelectric coefficients d_33_, k_p_, Qm and g_33_ for different *x* ratios of NKLN + *x* wt.% ZnO (*x* = 0 to 0.3). The experimental results indicate that increasing ZnO content achieves the optimal values of d33 and k_p_ while the dielectric constant εr is reduced. At *x* = 0.1, the material exhibits the best piezoelectric properties with d_33_ = 147, k_p_ = 0.44 and g_33_ = 40. This is primarily due to the coexistence of O-T phase transitions and improved density, which enhance the piezoelectric properties. However, when ZnO is added to *x* = 0.2, d_33_ and k_p_ decrease, consistent with the ferroelectric analysis. The increase in Qm with excessive ZnO addition suggests that excessive ZnO causes NKLN to act as a hardening agent, reducing d_33_ and k_p_. The detailed piezoelectric properties of the proposed samples are summarized in Table 1.

**Figure 7:**
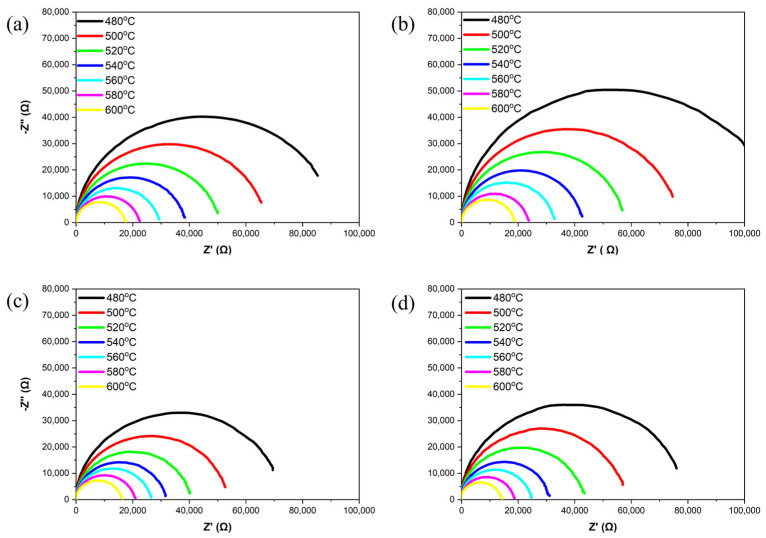
Complex impedance spectra of NKLN + *x* wt.% ZnO (*x* = 0 to 0.3) ceramics at temperatures from 480 °C to 600 °C: (**a**) *x* = 0; (**b**) *x* = 0.1; (**c**) *x* = 0.2; (**d**) *x* = 0.3.

Figure 7 shows the complex impedance spectra of the samples at different temperatures ranging from 480 °C to 600 °C. All samples exhibit a semi-circle in this temperature range, indicating that the grain and grain boundary have related characteristics [25]. As the ZnO content increases, the radius of the semi-circle reaches its maximum at *x* = 0.1 and then significantly decreases. This suggests that the optimal doping level is the ZnO content at *x* = 0.1. When ZnO is added in excess, the radius of the semi-circle decreases, likely due to a reduction in density, which leads to a decrease in insulation resistance.

**Figure 8:**
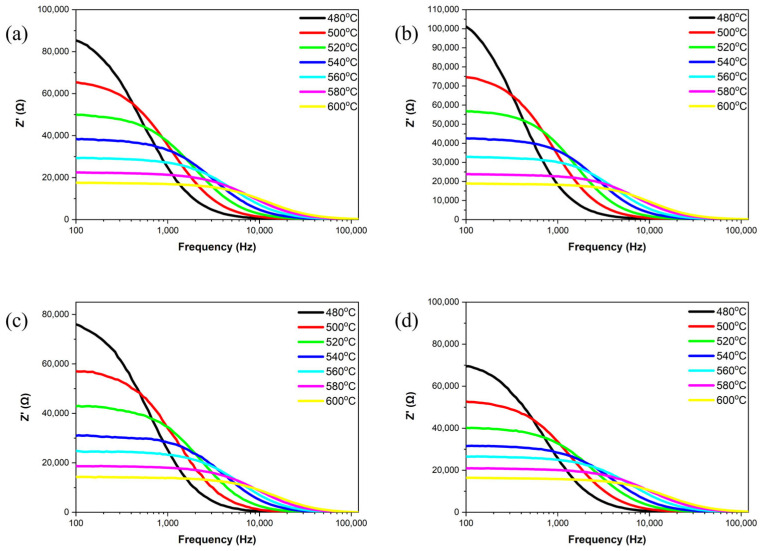
Frequency-dependent real part of impedance for NKLN + *x* wt.% ZnO (*x* = 0 to 0.3) ceramics at temperatures from 480 °C to 600 °C: (**a**) *x* = 0; (**b**) *x* = 0.1; (**c**) *x* = 0.2; (**d**) *x* = 0.3.

**Figure 9:**
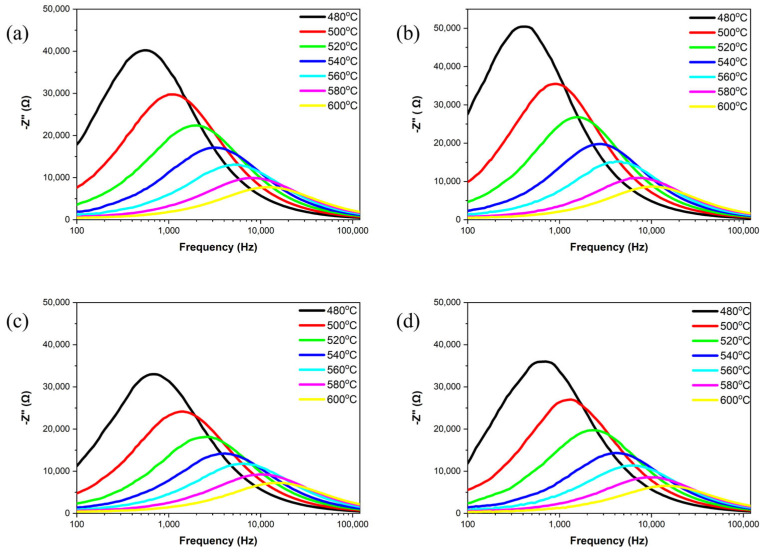
Frequency-dependent imaginary part of impedance for NKLN + *x* wt.% ZnO (*x* = 0 to 0.3) ceramics at temperatures from 480 °C to 600 °C: (**a**) *x* = 0; (**b**) *x* = 0.1; (**c**) *x* = 0.2; (**d**) *x* = 0.3.

Figure 8 and Figure 9 investigate the real part of impedance (Z′) and the imaginary part of impedance (Z″) as a function of frequency for all the samples at temperatures ranging from 480 °C to 600 °C. Within the 100 Hz to 100 kHz frequency range, the samples exhibit frequency dispersion and impedance relaxation phenomena with increasing temperature. As the frequency increases, space charge is released at high frequencies, and both the real and imaginary parts of impedance gradually converge to stable values [26,27]. This occurs because, at high frequencies, the charge carriers in the samples have enough energy to overcome potential barriers, resulting in reduced impedance in the high-frequency range [28]. This behavior demonstrates the negative temperature coefficient of the resistance characteristics of the samples, similar to that of semiconductors. During high-temperature sintering, the loss of oxygen ions leads to the semiconductor-like properties of the ceramics [27]. Peaks in the imaginary part of impedance (Z″) reflect the material’s temperature-dependent relaxation phenomena. As temperature increases, the intensity of these peaks decreases, broadens and shifts towards higher frequencies, indicating reduction relaxation times [29]. Additionally, the relaxation process of charge carriers and the frequency shift of the peaks are temperature-dependent, suggesting a reduction in the average relaxation time.

**Figure 10:**
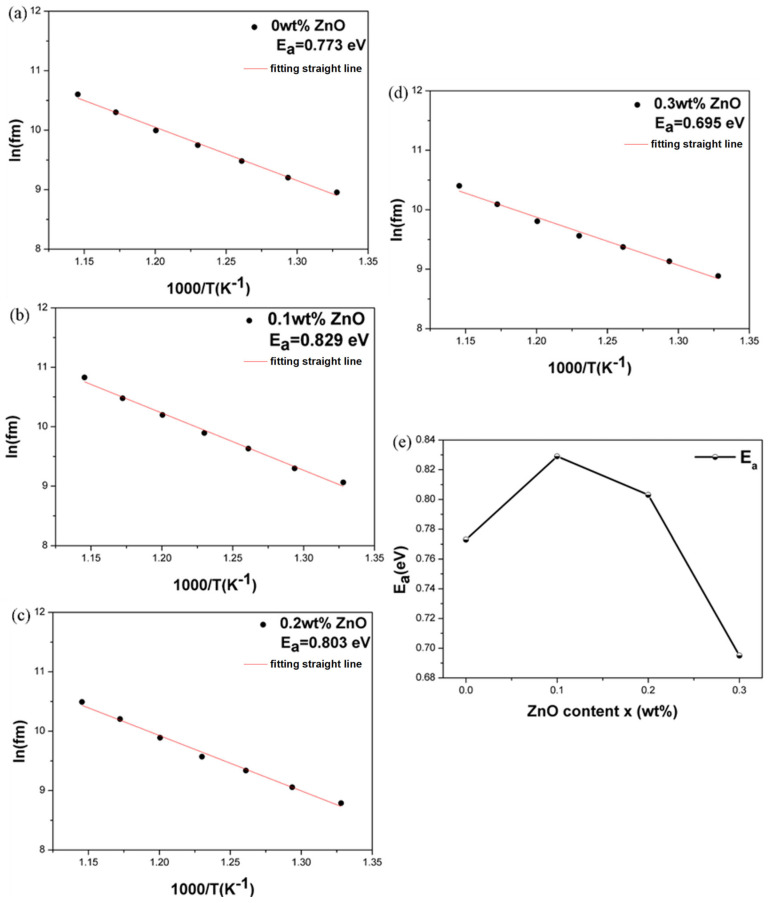
Variation in ln(f_m_) as a function of the inverse temperature (1000/T) for NKLN + *x* wt.% ZnO (*x* = 0 to 0.3) ceramics at temperatures from 480 °C to 600 °C: (**a**) *x* = 0; (**b**) *x* = 0.1; (**c**) *x* = 0.2; (**d**) *x* = 0.3; (**e**) activation energy (Ea).

Figure 10 shows the activation energy analysis of all the ceramics at temperatures from 480 °C to 600 °C. The activation energy (Ea) was calculated using the slope of the ln(fm) versus (1000/T) curve and the f_m_ represents the relaxation frequency at the measured temperature, with Ea values ranging from approximately 1 to 2 eV as summarized in Table 2. In perovskite structures, oxygen vacancy migration typically results in an activation energy of about 1 eV, while the conduction effects of relaxation materials show activation energies of approximately 0.36 to 0.67 eV. Activation energies for A-site cation migration are around 4 eV, while for B-site cation migration, they are about 14 eV [28]. After adding ZnO, the activation energy is approximately 0.8 eV, as the formation of oxygen vacancies is facilitated during high-temperature sintering. This effect can be confirmed using defect equations, suggesting that the change in activation energy is primarily due to oxygen vacancies rather than A- or B-site cation migration. At *x* = 0.1, the activation energy is at its highest, indicating that long-range charge carriers face significant potential barriers, which implies that *x* = 0.1 has the highest insulation resistance among all samples [30,31].

**Figure 11:**
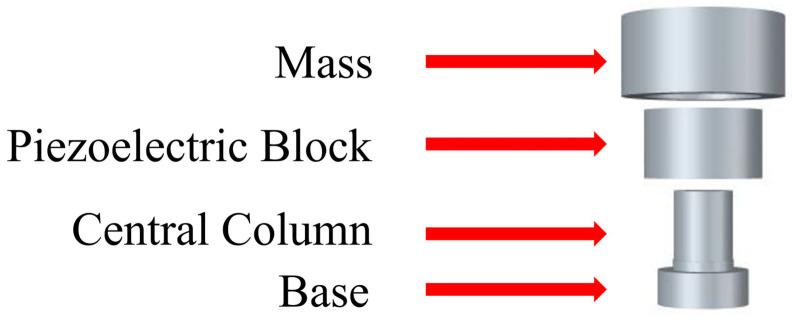
Exploded view of the shear-mode annular accelerometer structure.

**Figure 12:**
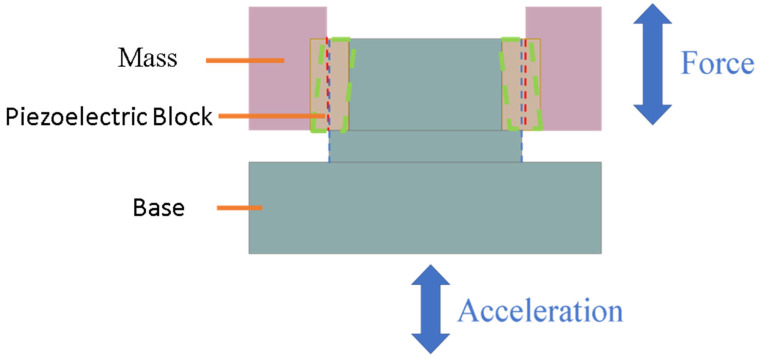
Schematic diagram of vibration in a shear-mode annular accelerometer.

Using the optimal piezoelectric coefficient g, we aim to fabricate annular ceramic components for accelerometer applications. There are few previous research studies about the piezoelectric shear mode accelerometers using high-g ceramics for high-bandwidth applications. ANSYS software is used to design a shear-mode accelerometer with the structure as shown in Figure 11. Furthermore, this design minimizes the impact of mechanical or environmental vibrations introduced by the base, enhancing resonance frequency and noise resistance. A higher g value indicates that the piezoelectric material can generate a substantial charge with minimal mechanical stress or strain, thus offering better performance in shear accelerometers. Figure 12 shows the structural design of the shear-mode annular piezoelectric accelerometer. When the base is subjected to acceleration and vertical displacement, it causes relative displacement between the base and the mass block, deforming the piezoelectric element and generating an electrical output. Suppose the central diameter of the mass block is smaller than the diameter of the second layer of the central column. In that case, the accelerometer will transition from shear deformation to purely compressive deformation. When the base moves up and down due to acceleration, there is a relative displacement between the base and the mass, which causes a deformation of the piezoelectric block (the green part is the hypothetical deformation line).The red and blue lines in the diagram illustrate the conditions for deformation: the blue line indicates that the base only drives half of the piezoelectric block, while the red line indicates that the displacement of the mass must not be the same as that of the base, otherwise the piezoelectric block will only be subjected to compression and will not be able to produce the desired shear deformation.

**Figure 13:**
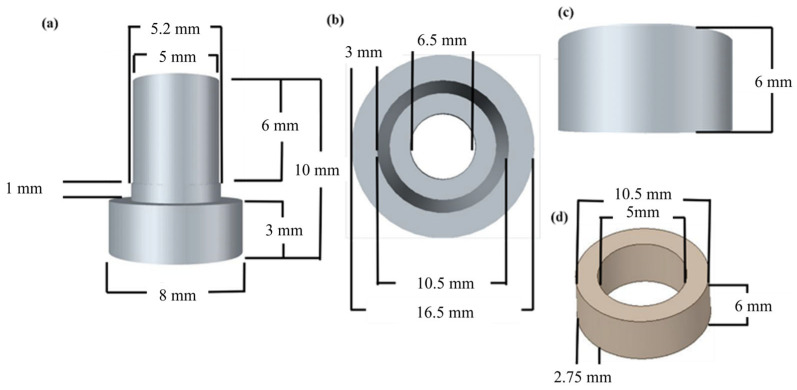
Dimensions of the shear-mode annular piezoelectric accelerometer: (**a**) base; (**b**) bottom view of the mass block; (**c**) cross-sectional view of the mass block; (**d**) annular piezoelectric element.

Figure 13 shows the construction of a shear-mode accelerometer made from the optimized process parameters. The annular ceramic has an outer diameter of approximately 10.5 mm, a central hole diameter of about 5 mm, a height of 6 mm and a thickness of 2.75 mm. The base has a physical size of a circular base with an 8 mm edge. The first-layer support has an outer diameter of approximately 5 mm and a height of 6 mm, while the second-layer support has an outer diameter of about 5.2 mm and a height of 1 mm. The mass block has a physical size with an outer diameter of about 2 mm, a central hole diameter of approximately 6.5 mm, a height of 6 mm and a thickness of 3 mm. The annular ceramic is first attached to the supports using AB silver adhesive. The mass block is then placed on top, with the top connected via wiring and the bottom electrode connected through the base, forming a parallel structure. Additionally, a lead wire is extended from the base for measurement purposes.

Table 3 shows the mechanical and piezoelectric coefficient data for the piezoelectric elements, as provided in Table 1, which were input into the ANSYS 2024 R1 software for analysis.

**Figure 14:**
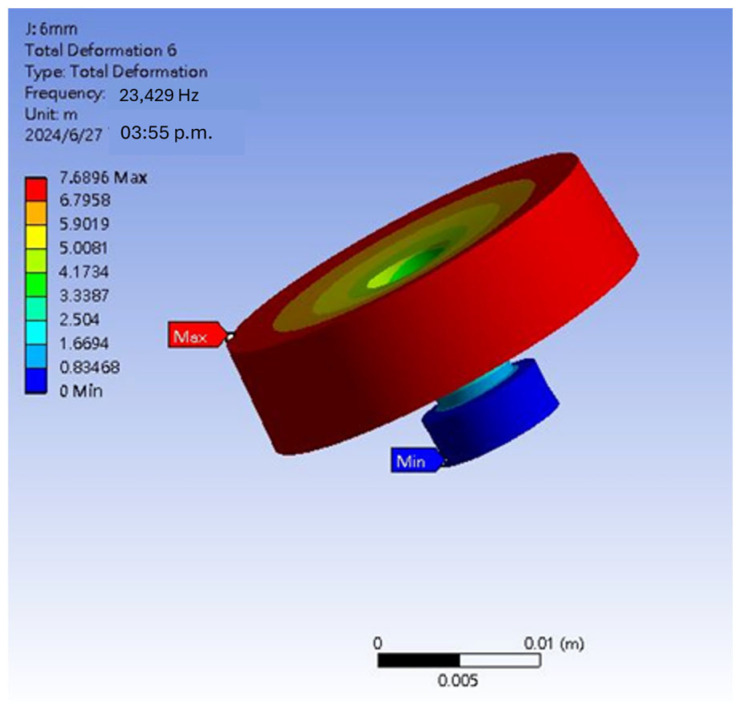
Vibration simulation of the shear-mode annular accelerometer.

**Figure 15:**
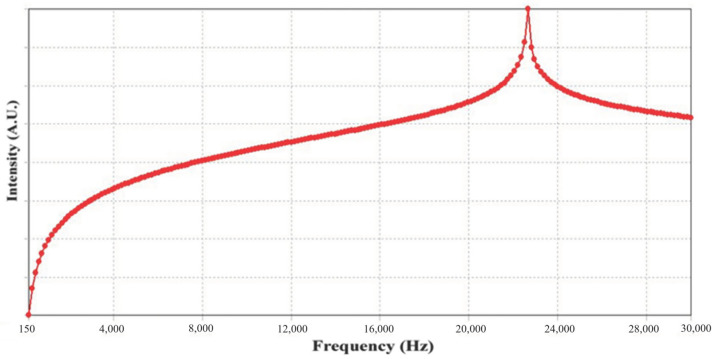
Frequency response simulation of the shear-mode annular accelerometer.

Figure 14 and Figure 15 show the vibration simulation of the shear-mode annular accelerometer. The base is fixed as a reference to simulate the strain of the component. Due to the accelerometer’s characteristics being measured in the vertical direction due to acceleration effects, the measurement is adjusted to simulate longitudinal vibration modes in the vertical plane to determine the resonance frequency. The figures reveal that when the accelerometer undergoes vertical oscillations, the resonance mode shows a resonance frequency of approximately 23 kHz, with the stress intensity on the left.

**Figure 16:**
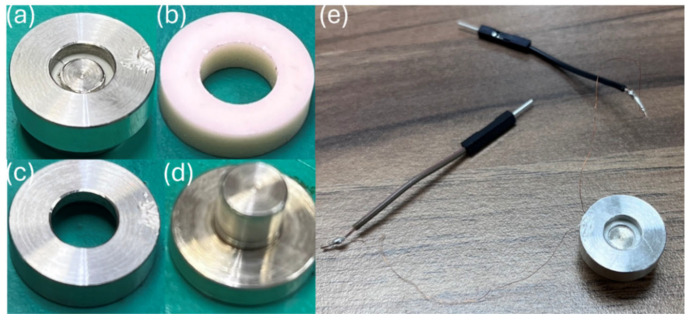
Shear-mode annular piezoelectric accelerometer physical images: (**a**) complete accelerometer; (**b**) piezoelectric element; (**c**) mass block; (**d**) base; (**e**) assembly diagram.

**Figure 17:**
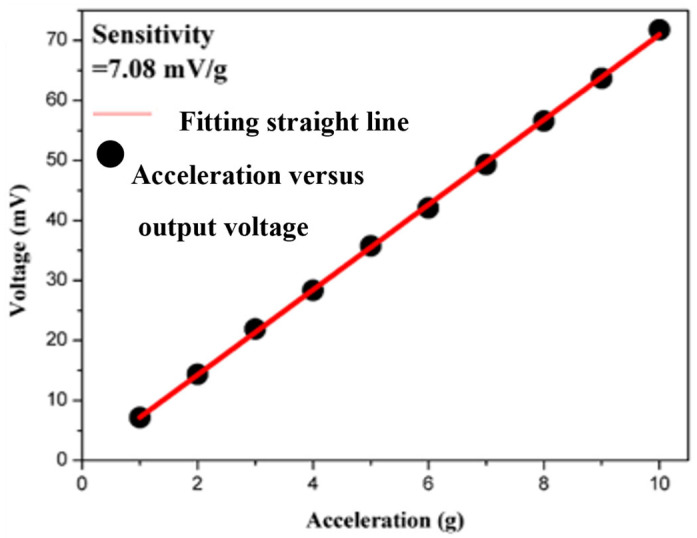
Acceleration versus output voltage for the shear-mode annular piezoelectric accelerometer.

Figure 16 shows the actual fabricated accelerometer. For accelerometers, the range of g values (where g = 9.8 m/s^2^) is a defining characteristic that needs to be specified. The measurement method involves mounting the accelerometer on an oscillator and applying different acceleration amplitudes at a fixed frequency. Within the measurement range, the results are assessed for linearity; the linear region defines the usable acceleration range of the accelerometer. Figure 17 presents the acceleration versus output voltage plot for the shear-mode annular piezoelectric accelerometer, measured using the accelerometer analysis system. The data within the measurement range show linearity. However, due to the specifications of the oscillator used in this experiment, measurements beyond 10 g acceleration could not be performed, and the limit range has not been determined. Currently, the operational range of the accelerometer in this experiment is defined as up to 10 g. Additionally, as the applied acceleration increases, the output voltage rises from 7.01 mV to 70.21 mV. Linear regression indicates that the sensitivity is approximately 7.08 mV/g.

**Figure 18:**
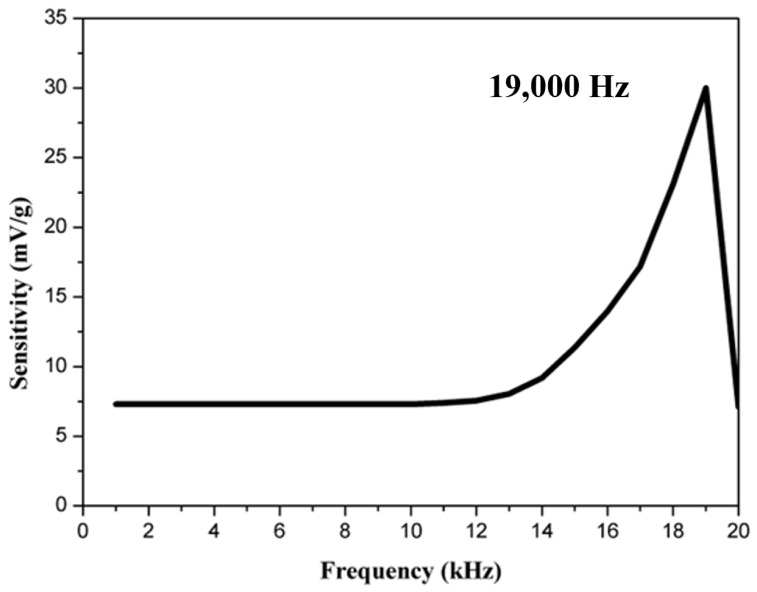
Frequency sweep results for the shear-mode annular piezoelectric accelerometer.

Frequency sweep-sensitivity measurement is a crucial and fundamental technique for assessing the bandwidth and sensitivity of an accelerometer. Figure 18 shows the frequency sweep results for the shear-mode annular piezoelectric accelerometer measured using the accelerometer analysis system. The frequency was swept from 100 Hz to 20 kHz, and the sensitivity increased with frequency, reaching its maximum value around 19 kHz, indicating the resonance frequency.

**Figure 19:**
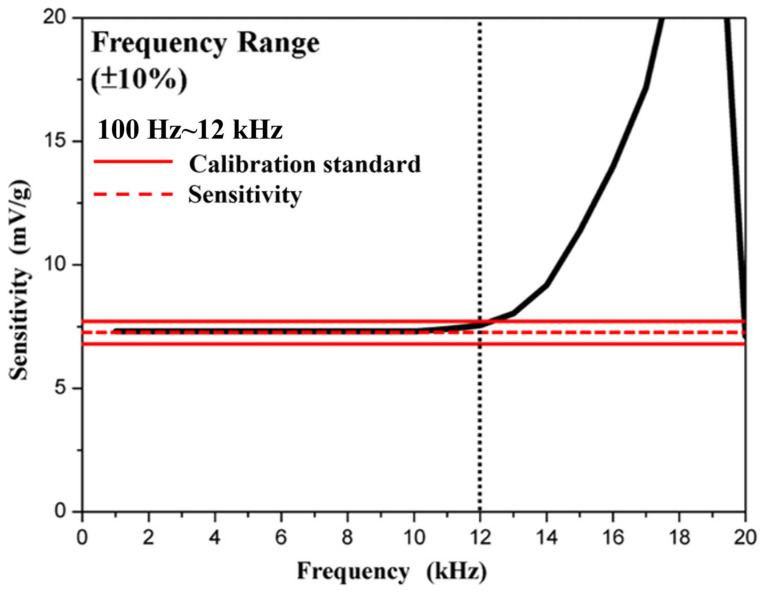
Frequency range measurement results for the shear-mode annular piezoelectric accelerometer. The full black line refers to the sensitivity of the accelerometer at different frequencies.

Figure 19 shows the frequency range measurement results. According to commercial sensor requirements, a frequency response within ±10% is used as a calibration standard (red line). Significant fluctuations are not allowed within this ±10% range to ensure stable measurement performance. The black dotted line is the focus of the red line and the black line, which refers to the maximum usable frequency within the calibration standard. This study sets the minimum frequency measurement at 100 Hz. The results indicate that the shear-mode accelerometer in this study has a usable frequency range from 100 Hz to 12 kHz, making it suitable for high-frequency vibration monitoring or analysis.

In the current research and commercialization, most shear-mode accelerometers use PZT as the piezoelectric material, with performance comparable to or better than the accelerometer developed in this study, as shown in Table 4. However, this study’s innovation lies in using lead-free NKN materials as a substitute for lead-based materials, enhancing performance through its piezoelectric properties. By utilizing NKLN and adding 0.1 wt.% ZnO, a shear-mode accelerometer was successfully fabricated with a resonance frequency of 19 kHz, a usable frequency range (±10%) from approximately 100 Hz to 12 kHz, a sensitivity of about 7.08 mV/g and a charge sensitivity of 13.2 pC/g.

## 4. Conclusions

In this study, using (Na_0.475_K_0.475_Li_0.05_)NbO_3_ as the base material, and adding ZnO to enhance its piezoelectric properties, resulted in g_33_ = 40 mV·m/N, k_p_ = 0.44, Qm = 89, ε_r_ = 406 and d_33_ = 147 pC/N. Utilizing the g_33_ property, a high-frequency shear-mode annular accelerometer was fabricated. ANSYS simulation software was used to design and simulate the shear-mode annular accelerometer, resulting in a resonance frequency of approximately 23 kHz. The actual accelerometer sensitivity is about 7.08 mV/g, the resonance frequency is 19 kHz and the usable frequency range (±10%) is from 100 Hz to 12 kHz. These characteristics demonstrate that this accelerometer, made from lead-free materials, can be applied to monitoring rotating machinery, predicting mechanical failures and enabling predictive maintenance, among other applications. It meets the demands of commercial use, particularly excelling in environmental performance and cost-effectiveness.

## Figures and Tables

**Table 1:** Piezoelectric properties of NKLN + *x* wt.% ZnO (*x* = 0 to 0.3).

*x* wt.%ZnO	*x* = 0	*x* = 0.1	*x* = 0.2	*x* = 0.3
**Z_min_ (ohm)**	133	139	124	276
**d_33_ (pC/N)**	127	147	115	94
***g*_33_ (mV·m/N)**	31	40	33	27
**k_p_**	0.39	0.44	0.38	0.35
**Q_m_**	92	88	96	114
**ε_r_**	458	406	386	351
**AGS (μm)**	1.87	2.82	3.76	4.16

**Table 2:** Variation in activation energy of NKLN + *x* wt.% ZnO (*x* = 0 to 0.3).

Compositions (*x*)	0	0.1	0.2	0.3
E_a_	0.773	0.829	0.803	0.695

**Table 3:** Piezoelectric element mechanical data.

Density (g/cm^3^)	4.373
Poisson’s Ratio	0.3
Young‘s Modulus (Pa)	1.475 × 10^11^

**Table 4:** Comparison table of performance between related research and commercial shear-mode piezoelectric accelerometers.

Materials	Mode	Sensitivity (mV/g)	Frequency Resonance (kHz)	Ref.	Manufacturer
PZT(KA)	Shear	3	10	[32]	Unictron Technologies Corporation
PZT-5H	Shear	1.6	6	[33]	Piezo Systems
Model 7104A Accelerometer	Shear	5	10	[34]	System Access Co., Ltd.
KS943L Accelerometer	Shear	14	19	[35]	System Access Co., Ltd.
KS94C10 Accelerometer	Shear	10	16	[35]	System Access Co., Ltd.
NKLN + 0.1 wt.% ZnO	Shear	7.08	19	This work	

## Data Availability

The original contributions presented in this study are included in the article. Further inquiries can be directed to the corresponding author.

## References

[1] Ding Y., Wang Y., Liu W., Pan Y., Yang P., Meng D., Zheng T., Wu J. (2024). Shear-structured piezoelectric accelerometers based on KNN lead-free ceramics for vibration monitoring. J. Mater. Chem. C.

[2] Jaffe H. (1958). Piezoelectric ceramics. J. Am. Ceram. Soc..

[3] Hu S., Luo C., Li P., Hu J., Li G., Jiang H., Zhang W. (2017). Effect of sintered temperature on structural and piezoelectric properties of barium titanate ceramic prepared by nano-scale precursors. J. Mater. Sci. Mater. Electron..

[4] Kobayashi K., Doshida Y., Mizuno Y., Randall C.A. (2012). A route forwards to narrow the performance gap between PZT and lead-free piezoelectric ceramic with low oxygen partial pressure processed (Na_0.5_ K_0.5_) NbO_3_. J. Am. Ceram. Soc..

[5] Huan Y., Wang X., Wei T., Xie J., Ye Z., Zhao P., Li L. (2017). Defect engineering of High-performance potassium sodium niobate piezoelectric ceramics sintered in reducing atmosphere. J. Am. Ceram. Soc..

[6] Mgbemere H.E., Herber R.-P., Schneider G.A. (2009). Effect of MnO_2_ on the dielectric and piezoelectric properties of alkaline niobate based lead free piezoelectric ceramics. J. Eur. Ceram. Soc..

[7] Wang Z., Huan Y., Wang H., Qiu Y., Feng Y., Wei T., Yang C. (2021). The optimal sintering atmosphere and defect structure of CuO-doped NKN-based ceramic with p/n-type conduction mechanism. J. Mater. Sci. Mater. Electron..

[8] Pang X., Qiu J., Zhu K., Shao B. (2011). Influence of sintering temperature on piezoelectric properties of (K_0.4425_Na_0.52_Li_0.0375_)(Nb_0.8925_Sb_0.07_Ta_0.0375_)O_3_ lead-free piezoelectric ceramics. J. Mater. Sci. Mater. Electron..

[9] Zuo R., Ye C., Fang X. (2008). Na_0. 5_K_0. 5_NbO_3_–BiFeO_3_ lead-free piezoelectric ceramics. J. Phys. Chem. Solids.

[10] Li P., Zhai J., Shen B., Zhang S., Li X., Zhu F., Zhang X. (2018). Ultrahigh piezoelectric properties in textured (K, Na) NbO_3_-based lead-free ceramics. Adv. Mater..

[11] Feng W., Du H., Chen C., Huang Y. (2016). Electric-Field-Driven Phase Transition Process in (K, Na, Li)(Nb, Ta, Sb) O_3_ Lead-Free Piezoceramics. J. Am. Ceram. Soc..

[12] Kim J., Ji J.-H., Shin D.-J., Koh J.-H. (2018). Improved Li and Sb doped lead-free (Na, K) NbO_3_ piezoelectric ceramics for energy harvesting applications. Ceram. Int..

[13] Lin D., Kwok K.W., Lam K.H., Chan H. (2008). Phase structure and electrical properties of K_0. 5_Na_0.5_ (Nb_0.94_Sb_0.06_) O_3_-LiTaO_3_ lead-free piezoelectric ceramics. J. Phys. D Appl. Phys..

[14] Su H.-H., Hong C.-S., Chen H.-R., Juang Y.-D., Tsai C.-C., Chu S.-Y. (2018). Phase Structure Transformations and Electrical Properties of (Na_0. 52_K_0. 4425_)(Nb_0. 8925_Sb_0. 07_) O_3_–0.0375 LiTaO_3_ Ceramics According to Sintering Temperature. ECS J. Solid State Sci. Technol..

[15] Zhang Y.-X., Chu S.-Y., Tsai C.-C., Hong C.-S., Huang J.-H., Wu Q.-Y., Chen Y.-A. (2025). The LiF doping effects on the microstructure, ferroelectric and electrocaloric properties of (Na, K, Li)(Nb, Sb, Ta)O_3_-based ceramics sintered in air atmosphere and reduced atmosphere. Mater. Sci. Eng. B.

[16] Weng C.-M., Tsai C.-C., Sheen J., Hong C.-S., Chu S.-Y., Chen Z.-Y., Su H.-H. (2017). Low-temperature–sintered CuF_2_-doped NKN ceramics with excellent piezoelectric and dielectric properties. J. Alloys Compd..

[17] Yang S.-L., Tsai C.-C., Liou Y.-C., Hong C.-S., Chen H.-C., Chu S.-Y. (2012). Differences Between Copper-Oxide- and Zinc-Oxide-Doped Sodium Potassium Niobate Ceramics. J. Am. Ceram. Soc..

[18] Zhang J. (2019). Dielectric, Ferroelectric and Piezoelectric Properties of PZT Ceramics by ZnO Doping. Integr. Ferroelectr..

[19] Li J.-W., Liu Y.-X., Thong H.-C., Du Z., Li Z., Zhu Z.-X., Nie J.-K., Geng J.-F., Gong W., Wang K. (2020). Effect of ZnO doping on (K,Na)NbO_3_-based lead-free piezoceramics: Enhanced ferroelectric and piezoelectric performance. J. Alloys Compd..

[20] Lee M.-K., Kim B.-H., Lee G.-J. (2023). Lead-Free Piezoelectric Acceleration Sensor Built Using a (K, Na) NbO_3_ Bulk Ceramic Modified by Bi-Based Perovskites. Sensors.

[21] Azough F., Wegrzyn M., Freer R., Sharma S., Hall D. (2011). Microstructure and piezoelectric properties of CuO added (K, Na, Li)NbO_3_ lead-free piezoelectric ceramics. J. Eur. Ceram. Soc..

[22] Li E., Kakemoto H., Wada S., Tsurumi T. (2007). Influence of CuO on the structure and piezoelectric properties of the alkaline niobate-based lead-free ceramics. J. Am. Ceram. Soc..

[23] Rubio-Marcos F., Navarro-Rojero M.G., Romero J.J., Marchet P., Fernandez J.F. (2009). Piezoceramics properties as a function of the structure in the system (K, Na, Li)(Nb, Ta, Sb)O_3_. IEEE Trans. Ultrason. Ferroelectr. Freq. Control.

[24] Dai Y., Zhang X., Zhou G. (2007). Phase transitional behavior in K_0.5_Na_0.5_NbO_3_–LiTaO_3_ ceramics. Appl. Phys. Lett..

[25] Miah M., Khan M., Hossain A.A. (2016). Synthesis and enhancement of multiferroic properties of (x) Ba_0.95_Sr_0.05_TiO_3–_(1 − x) BiFe_0.90_Dy_0.10_O_3_ ceramics. J. Magn. Magn. Mater..

[26] Saidi M., Chaouchi A., d’Astorg S., Rguiti M., Courtois C. (2015). Dielectric, ferroelectrics properties and impedance spectroscopy analysis of the [(Na_0.535_ K_0.480_)_0.966_ Li_0.058_](Nb_0.90_ Ta_0.10_) O_3_-based lead-free ceramics. J. Adv. Dielectr..

[27] Adak M.K., Dhak D. (2019). Assessment of strong relaxation on BaTiO_3_ modified by Mn_2_^+^ and Pr_3_^+^, K^+^ at A- and B-site respectively. Mater. Res. Express.

[28] Steinsvik S., Bugge R., Gjønnes J., Taftø J., Norby T. (1997). The defect structufe of SrTi_1−x_FexO_3−y_ (x= 0–0.8) investigated by electrical conductivity measurements and electron energy loss spectroscopy (EELS). J. Phys. Chem. Solids.

[29] Liu Y., Du Y., Cheng C., Sun X., Jiang N., Wang J., Sun X. (2019). Dielectric and impedance spectroscopy analysis of lead-free (1-x)(K_0.44_Na_0.52_Li_0.04_)(Nb_0.86_Ta_0.10_Sb_0.04_) O_3−x_BaTiO_3_ ceramics. Ceram. Int..

[30] Tsai C.-C., Liao W.-H., Chu S.-Y., Hong C.-S., Yu M.-C., Wei Z.-Y., Lin Y.-Y. (2021). Effect of BaTiO_3_ templates on the electrical characteristics of (Ba, Ca)(Ti, Sn, Hf)-based ceramics under the reducing atmosphere for actuator applications. Ceram. Int..

[31] Tsai C.-C., Liao W.-H., Chu S.-Y., Hong C.-S., Yu M.-C., Lin Y.-Y., Wei Z.-Y. (2021). Investigation of the piezoelectric and anti-reduction properties of (Ba, Ca)(Ti, Sn, Hf) textured ceramics prepared under low oxygen partial pressure conditions at low sintering temperatures. J. Eur. Ceram. Soc..

[32] Li D.-Y. (2019). Shear Accelerometers Design and Application on High Speed Spindle. Master’s Thesis.

[33] Mo Y.-C., Su K.-Y., Kang W.-B., Chen L.-B., Chang W.-J., Liu Y.-H. An FFT-based high-speed spindle monitoring system for analyzing vibrations. Proceedings of the 2017 Eleventh International Conference on Sensing Technology (ICST).

[34] Measurement Specialties, Inc (2012). Model 7104A Accelerometer. http://www.systemaccess.com.tw/upload/web/Product/Accelerometer/PDF/AC1/(7104A)-7104A_Accelerometer.

[35] KS943 Series Accelerometer, Metra Meß-und Frequenztechnik in Radebeul e.K. https://www.systemaccess.com.tw/upload/web/Download/MMF/1-8-1_KS943B10_KS943B100_KS943L.pdf.2019.

